# Amino Acid Mutations A286V and T437M in the Nucleoprotein Attenuate H7N9 Viruses in Mice

**DOI:** 10.1128/JVI.01530-19

**Published:** 2020-01-06

**Authors:** Shujie Ma, Bo Zhang, Jianzhong Shi, Xin Yin, Guangwen Wang, Pengfei Cui, Liling Liu, Guohua Deng, Yongping Jiang, Chengjun Li, Hualan Chen

**Affiliations:** a State Key Laboratory of Veterinary Biotechnology, Harbin Veterinary Research Institute, Chinese Academy of Agricultural Sciences, Harbin, People’s Republic of China; Cornell University

**Keywords:** H7N9, genetic basis, influenza, virulence

## Abstract

The low-pathogenic H7N9 influenza viruses that emerged in 2013 acquired an insertion of four amino acids in their hemagglutinin cleavage site and thereby became highly pathogenic to chickens in 2017. Previous studies indicated that these highly pathogenic H7N9 viruses are virulent in chickens but have distinct pathotypes in mice. A/chicken/Guangdong/SD098/2017 (CK/SD098) is avirulent, with a 50% mouse lethal dose (MLD_50_) of >7.5 log_10_ 50% egg infectious dose (EID_50_), whereas A/chicken/Hunan/S1220/2017 (CK/S1220) is virulent in mice, with an MLD_50_ of 3.2 log_10_ EID_50_. In this study, we explored the genetic determinants that contribute to the difference in virulence between these two H7N9 viruses by generating a series of reassortants and mutants in the CK/S1220 virus background and testing their virulence in mice. We found that the reassortant CK/1220-SD098-NP, carrying the nucleoprotein (NP) of CK/SD098, was avirulent in mice, with an MLD_50_ of >10^7.5^ EID_50_. The NPs of these two viruses differ by two amino acids, at positions 286 and 437. We further demonstrated that the amino acid mutations A286V and T437M of NP independently slowed the process of NP import to and export from the nucleus and thus jointly impaired the viral life cycle and attenuated the virulence of these H7N9 viruses in mice. Our study identified new virulence determinants in NP and provided novel targets for the development of live attenuated vaccines and antiviral drugs against influenza viruses.

**IMPORTANCE** The H7N9 influenza viruses that emerged in China in 2013 have caused over 1,500 human infections, with a mortality rate of nearly 40%. The viruses were initially low pathogenic but became highly pathogenic in chickens at the beginning of 2017 and caused severe disease outbreaks in poultry. Several studies suggested that the highly pathogenic H7N9 viruses have increased virulence in mammals; however, the genetic basis of the virulence of H7N9 viruses in mammals is not fully understood. Here, we found that two amino acids, 286A and 437T, in NP are prerequisites for the virulence of H7N9 viruses in mice and the mutations A286V and T437M collectively eliminate the virulence of H7N9 viruses in mice. Our study further demonstrated that the virulence of influenza viruses is a polygenic trait, and the newly identified virulence-related residues in NP may provide new targets for attenuated influenza vaccine and antiviral drug development.

## Abstract (precis)

The H7N9 influenza viruses that emerged in China in 2013 have caused over 1,500 human infections, with a mortality rate of nearly 40%. The viruses were initially low pathogenic but became highly pathogenic in chickens at the beginning of 2017 and caused severe disease outbreaks in poultry. Several studies suggested that the highly pathogenic H7N9 viruses have increased virulence in mammals; however, the genetic basis of the virulence of H7N9 viruses in mammals is not fully understood. Here, we found that two amino acids, 286A and 437T, in NP are prerequisites for the virulence of H7N9 viruses in mice and the mutations A286V and T437M collectively eliminate the virulence of H7N9 viruses in mice. Our study further demonstrated that the virulence of influenza viruses is a polygenic trait, and the newly identified virulence-related residues in NP may provide new targets for attenuated influenza vaccine and antiviral drug development.

## INTRODUCTION

Influenza A virus is a negative-sense, single-stranded, eight-gene-segmented RNA virus that belongs to the family *Orthomyxoviridae*. The eight gene segments encode basic polymerase 2 (PB2), basic polymerase 1 (PB1), acidic polymerase (PA), hemagglutinin (HA), nucleoprotein (NP), neuraminidase (NA), matrix (M), and nonstructural protein (NS), and each of them encodes one to four proteins (1). On the basis of the antigenic differences of the two surface glycoproteins, HA and NA, influenza A viruses are divided into different subtypes. Viruses of 16 different HA subtypes and nine different NA subtypes have been identified in aquatic birds, which are the natural hosts of influenza A viruses.

Influenza A viruses are important pathogens that affect both human and animal health. The virulence of influenza viruses varies among strains, and the same virus may have different pathotypes in different animals. Numerous studies have been performed to identify the key genetic determinants that contribute to influenza virus lethality. Extra amino acids inserted into the HA cleavage site usually turn low-pathogenic H5 and H7 avian influenza viruses into highly pathogenic strains in poultry (2–7). Kawaoka et al. reported that the lack of a glycosylation site at position 11 of HA increased the virulence of H5N2 virus in chickens (8), and Zhao et al. reported that the G158N mutation introduced *N*-linked glycosylation at positions 158 to 160 in HA and thereby enhanced the virulence of H5N1 virus in mice (9). The single-amino-acid mutations E627K and D701N in the PB2 protein have been reported to increase the virulence of different avian influenza viruses in mice, ferrets, and humans (2, 10–14). Feng et al. demonstrated that the G622D mutation in PB1 impaired the binding of PB1 to viral RNA, thereby dramatically decreasing the polymerase activity and attenuating the virulence of H5N1 avian influenza virus in mice (15). The amino acid mutations S224P, N383D, and I353R in PA increased the virulence of H5N1 influenza virus in mice and ducks (16–18). The amino acid mutations M105V, I109T, and A184K in NP increased the virulence of H5N1 virus in chickens (19–21), whereas the K470R mutation in NP increased the virulence of H5N1 virus in mice (22). Two amino acid mutations in M1, N30D and T215A, synergistically increased the virulence of H5N1 virus in mice (23). Certain amino acids or motifs in the NS1 protein contribute to the virulence of influenza virus in chickens and mice by undermining the antiviral immune response of the host (24–27). These findings indicate that multiple genetic factors affect the virulence of influenza viruses.

The H7N9 viruses that emerged in China in 2013 have caused 1,568 human infections, and nearly 40% of the human cases were fatal (28) (note that different lineages of H7N9 influenza viruses have been detected in different countries and the H7N9 viruses we refer to in this text all belong to the China 2013 Anhui-like lineage). The viruses were initially low pathogenic in chickens but, after 4 years of circulation in nature, a few strains isolated from chickens in Guangdong Province in 2017 were found to have acquired 12 extra nucleotides, encoding four amino acids in the cleavage site of HA, and had become highly pathogenic in chickens (2, 29–33) (note that “highly pathogenic” in this text refers to the virulence of the virus in chickens). The H7N9 highly pathogenic viruses have increased virulence in humans (2, 33, 34) and could become highly lethal in mice and highly transmissible in ferrets after replication in ferrets or humans upon obtaining the PB2 627K or 701N mutation (2, 34). We previously found that naturally isolated highly pathogenic H7N9 viruses from chickens exhibit different pathotypes in mice (30). In the present study, we selected two viruses, namely, A/chicken/Hunan/S1220/2017(H7N9) (CK/S1220) and A/chicken/Guangdong/SD098/2017(H7N9) (CK/SD098), that are genetically highly similar but differ in their virulence in mice to investigate the genetic basis of their virulence difference and to explore the underlying mechanism.

## RESULTS

### H7N9 avian influenza viruses have different lethality in mice.

**FIG 1:**
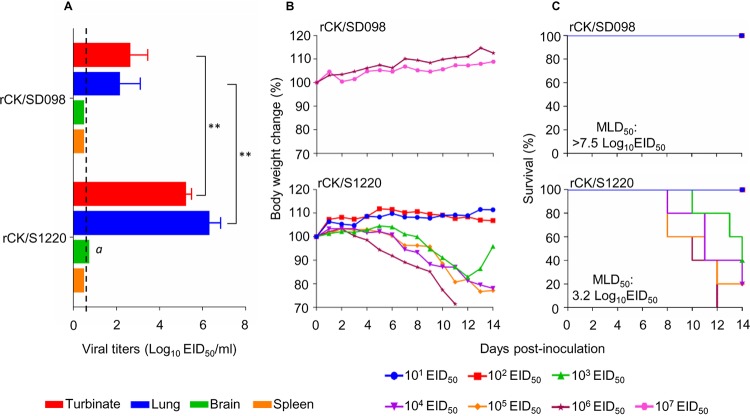
Replication and virulence of rCK/SD098 and rCK/S1220 viruses in BALB/c mice. (A) Virus replication titers in the indicated organs of mice after inoculation with 10^6^ EID_50_ of different viruses. Data shown are the mean virus titers ± standard deviation (*n* = 3), except for the values labeled *a*, indicating that virus was detected only in the organs of 2 mice. The dashed lines indicate the lower limit of virus detection. Virus titers of mice were statistically analyzed by using the one-tailed unpaired *t* test. **, *P *< 0.01. (B) Body weight changes in mice infected with the indicated viruses. (C) MLD_50s_ values for mice infected with the indicated viruses.

Our previous study indicated that CK/SD098 was not lethal in mice at a dose as high as 10^6^ times the 50% egg infectious dose (EID_50_), whereas the 50% mouse lethal dose (MLD_50_) of the CK/S1220 virus was 3.2 log_10_ EID_50_ (30). In this study, we rescued these two viruses by reverse genetics and designated them rCK/SD098 and rCK/S1220, respectively. The replication and virulence of these two rescued viruses were compared in mice. As shown in Fig. 1, rCK/S1220 replicated efficiently in the nasal turbinates and lungs of mice; virus was also detected in the brains of 2 of the 3 inoculated mice (Fig. 1A). In contrast, the rCK/SD098 virus replicated only in the nasal turbinates and lungs, with titers significantly lower than those in rCK/S1220-infected mice (Fig. 1A). To assess virulence, we inoculated groups of 5 mice intranasally with 10^6.0^ to 10^7.0^ EID_50_ of rCK/SD098 or 10^1.0^ to 10^6.0^ EID_50_ of rCK/S1220 and observed the animals for 2 weeks. As shown in Fig. 1B, none of the mice died in the rCK/SD098-inoculated groups, even in the group inoculated with 10^7.0^ EID_50_; therefore, the MLD_50_ for rCK/SD098 was >7.5 log_10_ EID_50_. Mice inoculated with high doses of rCK/S1220 virus died, which yielded an MLD_50_ for rCK/S1220 of 3.2 log_10_ EID_50_. These results indicate that the virulence of rCK/SD098 and that of rCK/S1220 in mice differ by >10,000-fold.

### The NP protein of CK/SD098 attenuates the CK/S1220 virus in mice.

**FIG 2:**
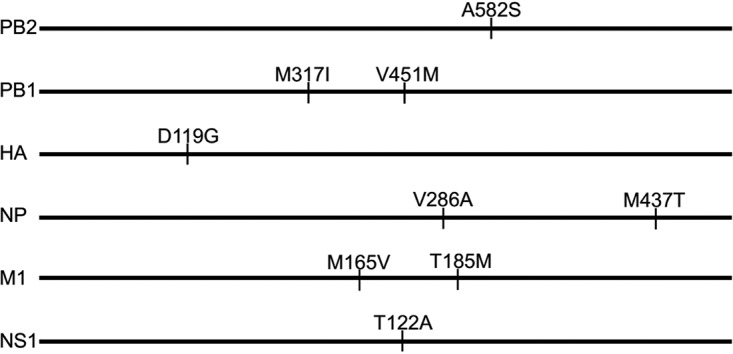
Amino acid differences between the CK/S1220 and CK/SD098 viruses. The amino acid differences between the two viruses are shown as single letters at the indicated positions. Each amino acid of CK/SD098 is shown before the number of the position, and each amino acid of CK/S1220 is shown after the number of the position. The amino acid at position 119 of HA is H3 numbering.

**FIG 3:**
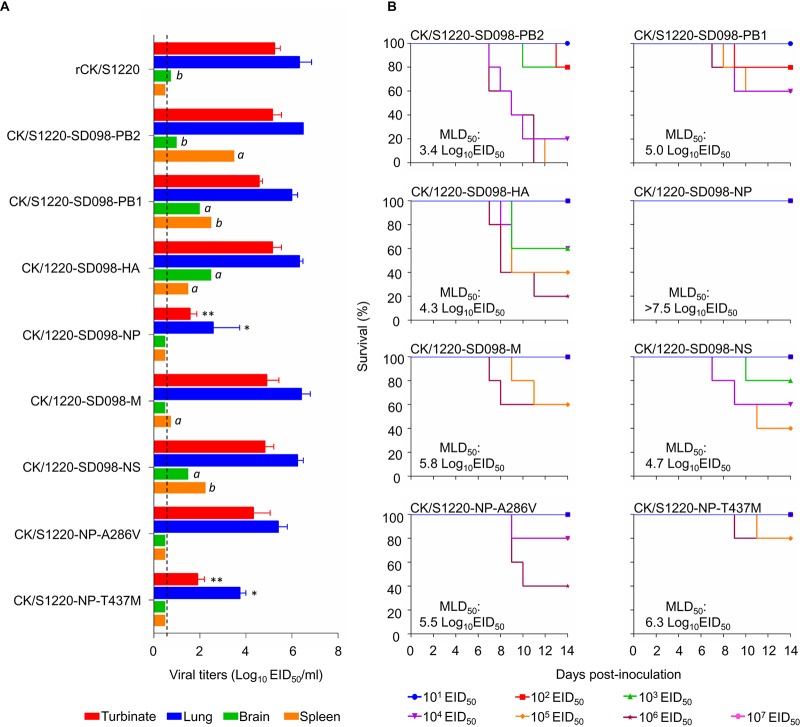
Replication and virulence of H7N9 viruses in BALB/c mice. (A) Virus replication titers in the indicated organs of mice after inoculation with 10^6^ EID_50_ of different viruses. Data shown are the mean virus titers ± standard deviation (*n* = 3), except for the values labeled *a* and *b*, indicating that virus was detected only in the organs of 1 mouse and 2 mice, respectively. The dashed lines indicate the lower limit of virus detection. The virus titers in mouse turbinates and lungs were compared with the rCK/S1220 titers, and statistical analysis was performed by using one-way ANOVA with GraphPad Prism 6 software (GraphPad Software Inc.). When one-way ANOVA was warranted, the *post hoc* analysis was performed using Dunnett’s test for multiple comparisons. *, *P *< 0.05; **, *P *< 0.01. (B) MLD_50s_ values for mice infected with the indicated viruses.

The CK/SD098 and CK/S1220 viruses differ only by nine amino acids located in six different proteins, i.e., PB2, PB1, HA, NP, M1, and NS1 (Fig. 2). To identify the genes that contribute to the virulence of these viruses in mice, we generated six reassortants by reverse genetics, using CK/S1220 as the backbone; each of the reassortants bears a CK/SD098 gene segment that differs from the CK/S1220 virus. We designated these reassortants CK/S1220-SD098-PB2, CK/S1220-SD098-PB1, CK/S1220-SD098-HA, CK/S1220-SD098-NP, CK/S1220-SD098-M, and CK/S1220-SD098-NS and tested their replication and virulence in mice. As shown in Fig. 3, CK/S1220-SD098-PB2, CK/S1220-SD098-PB1, CK/S1220-SD098-HA, CK/S1220-SD098-M, and CK/S1220-SD098-NS replicated in the nasal turbinates and lungs of mice, with titers comparable to those in rCK/S1220-inoculated mice. These viruses were also detected in the spleen and/or brain of 1 or 2 of the 3 inoculated mice (Fig. 3), indicating that certain gene constellations of the reassortants may play a role in altering tissue tropism. These five viruses were less lethal in mice than the rCK/S1220 virus, with MLD_50_ values ranging from 3.4 log_10_ EID_50_ to 5.8 log_10_ EID_50_ (Fig. 3). However, the virus titers in the nasal turbinates and lungs of the CK/S1220-SD098-NP-inoculated mice were significantly lower than those in the rCK/S1220-inoculated mice (Fig. 3), and all mice inoculated with CK/S1220-SD098-NP virus survived during the observation period, yielding an MLD_50_ of >7.5 log_10_ EID_50_ (Fig. 3). These results indicate that the NP protein of CK/SD098 completely attenuates the CK/S1220 virus in mice, although five other genes from the CK/SD098 virus also attenuate the CK/S1220 virus to different degrees.

There are only two amino acid differences in the NP protein of CK/S1220, compared with that of CK/SD098 (Fig. 2). To identify the contributions of these amino acids to the attenuation, we generated two mutants in the CK/S1220 backbone, designated CK/S1220-NP-A286V and CK/S1220-NP-T437M, and tested their replication and virulence in mice. CK/S1220-NP-A286V replicated efficiently in the turbinates and lungs of mice, with titers comparable to those in rCK/S1220-inoculated mice, whereas the virus titers in the lungs and nasal turbinates of CK/S1220-NP-T437M-inoculated mice were significantly lower than those in rCK/S1220-inoculated mice (Fig. 3). CK/S1220-NP-A286V and CK/S1220-NP-T437M were attenuated 200-fold and 1,259-fold, respectively, compared with the rCK/S1220 virus (MLD_50_ values of 5.5 log_10_ EID_50_ versus 3.2 log_10_ EID_50_ and 6.3 log_10_ EID_50_ versus 3.2 log_10_ EID_50_, respectively) (Fig. 3). These results indicate that the amino acid mutations A286V and T437M in NP have synergistic effects on the attenuation of H7N9 virus in mice, although the amino acid at position 437 of NP plays a major role in this function.

### T437M of NP reduces the polymerase activity and eliminates the replication of the CK/S1220 virus *in vitro*.

**FIG 4:**
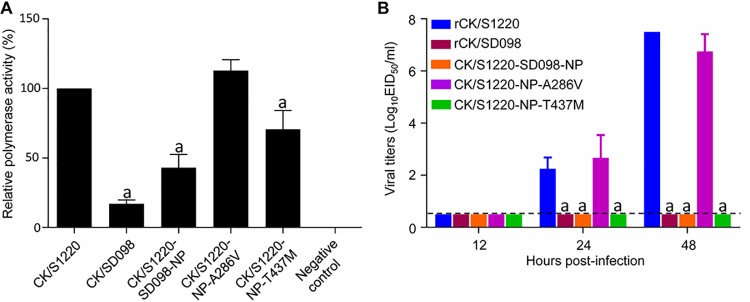
Polymerase activity and replication *in vitro* of H7N9 viruses bearing different NP mutations. (A) Polymerase activities of H7N9 wild-type viruses and NP mutants in a minigenome assay. The values shown are means ± standard deviations (*n* = 3) and are standardized to the activity of CK/S1220 (100%). (B) Multicycle replication of H7N9 viruses in MDCK cells. MDCK monolayers were inoculated with 10^3^ EID_50_ of the indicated viruses, and the culture supernatants were collected at the indicated times and then titrated in eggs. The dashed lines indicate the lower limit of virus detection. The virus titers were compared with those from rCK/S1220-infected cells. Statistical analysis was performed by using one-way ANOVA with GraphPad Prism 6 software. When one-way ANOVA was warranted, the *post hoc* analysis was performed using Dunnett’s test for multiple comparisons. a, *P *< 0.01.

NP is a component of the viral ribonucleoprotein (vRNP) complex, which plays an important role in influenza virus replication (35, 36). To investigate whether the mutations A286V and T437M in NP affect the polymerase activity of the H7N9 viruses, we tested the polymerase activities of the vRNP of CK/S1220 and its NP mutants by using a minigenome assay in 293T cells, as described previously (2). As shown in Fig. 4, the polymerase activity of the vRNP bearing the A286V mutation in NP was similar to that of the vRNP of the CK/S1220 virus, but the polymerase activity of the vRNP bearing the T437M mutation in NP or bearing the NP of the CK/SD098 virus (which has both the A286V and T437M mutations) was significantly lower than that of the vRNP of the CK/S1220 virus (Fig. 4A). To test whether these mutations in NP affected viral replication *in vitro*, we compared the multicycle growth of five viruses (rCK/S1220, rCK/SD098, CK/S1220-SD098-NP, CK/S1220-NP-A286V, and CK/S1220-NP-T437M) in Madin-Darby canine kidney (MDCK) cells. As shown in Fig. 4, rCK/S1220 and CK/S1220-NP-A286V replicated efficiently, but the replication of rCK/SD098, CK/S1220-SD098-NP, and CK/S1220-NP-T437M was below the limit of detection (Fig. 4B). These results indicate that the amino acid mutation T437M in NP decreases the polymerase activity and impairs the replication of the CK/S1220 virus *in vitro* but the amino acid mutation A286V in NP does not affect these functions.

### The A286V and T437M mutations in NP slow the nuclear import and export of the NP/vRNP of the CK/S1220 virus.

**FIG 5:**
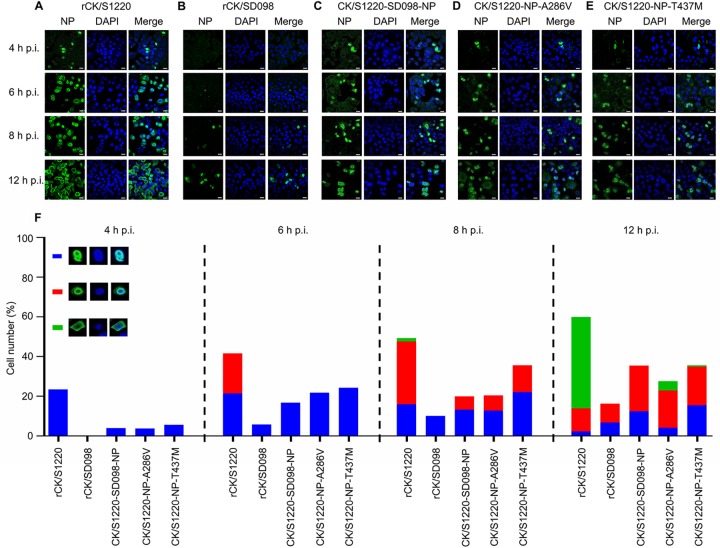
Quantitative analysis of NP localization in A549 cells infected with H7N9 viruses. (A to E) A549 cells were infected with 10^6^ EID_50_ of rCK/S1220 (A), rCK/SD098 (B), CK/S1220-SD098-NP (C), CK/S1220-NP-A286V (D), or CK/S1220-NP-T437M (E) viruses, and the localization of NP was determined by use of confocal microscopy at the indicated time points. (F) Quantitative analysis of NP localization in virus-infected cells was performed. The results shown were calculated from 300 cells. On the basis of the confocal microscopy findings in panels A, B, C, D, and E, the localization of NP was categorized into three types, i.e., clear nuclear localization (blue), simultaneous localization at the edge of the nucleus and the cytoplasm (red), and predominantly cytoplasmic localization (green).

The vRNP complex of influenza virus governs the synthesis of viral RNA and mRNA during virus replication, and this process occurs in the nucleus of the virus-infected cells. NP plays an important role in the nuclear import and export of the vRNP complex (37, 38). To investigate whether the mutations at positions 286 and 437 of NP affect the nuclear import and export of the vRNP, we observed the cellular distribution of NP in A549 cells that were infected with five different viruses, i.e., rCK/S1220, rCK/SD098, CK/S1220-SD098-NP, CK/S1220-NP-A286V, and CK/S1220-NP-T437M. In rCK/S1220-infected cells, the NP protein was detected in the nucleus of 23% of cells at 4 h postinfection (p.i.), as shown in Fig. 5. The NP protein was detected in the nucleus of 41% of cells at 6 h p.i., but the NP protein had moved to the edge of the nucleus in one-half of these cells (about 21%). By 8 h p.i., NP was detected in the nucleus of 16% of the cells, at the edge of the nucleus of 32% of the cells, and in the cytoplasm of 2% of the cells. By 12 h p.i., cells with NP in the nucleus, at the edge of the nucleus, and in the cytoplasm represented 2%, 12%, and 46%, respectively, of the total cells (Fig. 5A and F). However, the numbers of cells with NP in the nucleus at 4, 6, and 8 h p.i. for the other four viruses were notably lower than those for rCK/S1220-infected cells (Fig. 5B to E), and the percentages of cells with NP in the cytoplasm of the total cells infected with CK/SD098, CK/S1220-SD098-NP, CK/S1220-NP-A286V, and CK/S1220-NP-T437M were 0%, 0%, 5%, and 1%, respectively, at 12 h p.i. (Fig. 5F). These results indicate that both the A286V and T437M mutations in NP slow the process of the nuclear import and export of NP/vRNP in A549 cells and therefore may slow virus replication *in vivo*.

### The amino acid mutations A286V and T437M do not affect NP homo-oligomerization or the interactions between NP and importin α.

**FIG 6:**
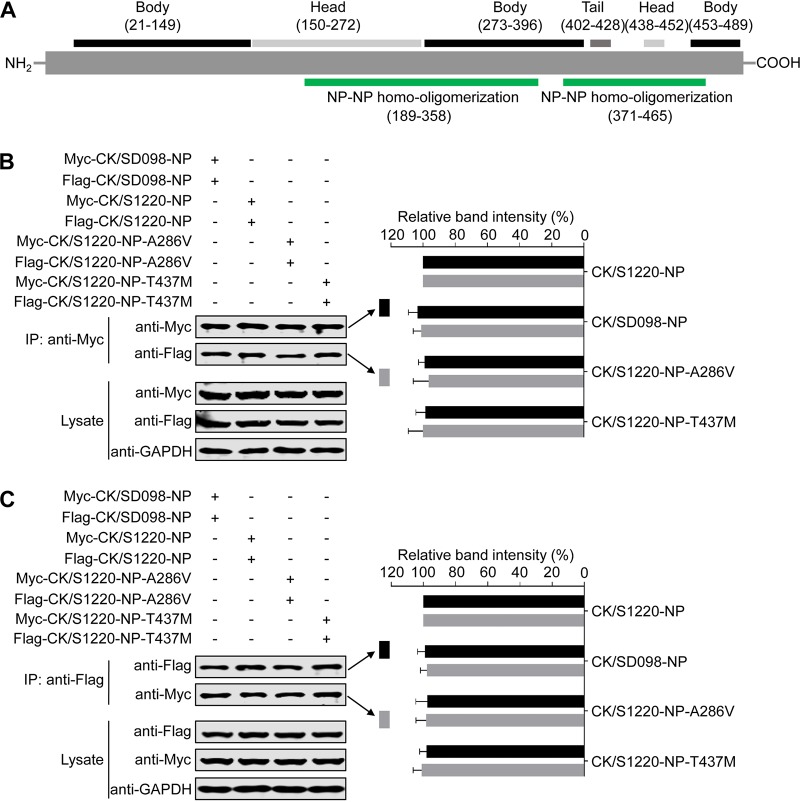
Effects of the amino acids at positions 286 and 437 of NP on NP-NP homo-oligomerization. (A) Schematic representation of influenza virus NP. (B and C) NP-NP homo-oligomerization assay results. NP-NP homo-oligomerization analysis was performed by transfecting 293T cells with Myc-tagged NP and Flag-tagged NP; at 36 h posttransfection, cell lysates were immunoprecipitated with an anti-Myc MAb, followed by Western blotting with MAbs against the Myc tag and the Flag tag (B), or the cell lysates were immunoprecipitated with an anti-Flag MAb, followed by Western blotting with MAbs against the Myc tag and the Flag tag (C). IP, immunoprecipitation. The band intensities of the Western blots from three assays were quantified by using ImageJ software and compared with the value of the CK/S1220-NP-transfected sample. Statistical analysis was performed by using one-way ANOVA with GraphPad Prism 6 software. When one-way ANOVA was warranted, the *post hoc* analysis was performed using Dunnett’s test for multiple comparisons; no statistically significant differences were detected. Only the band intensity values of the coimmunoprecipitated proteins are shown in panels B and C.

NP forms homo-oligomers to maintain the vRNP structure (39–43), and previous studies have identified two areas in NP (amino acids 189 to 358 and amino acids 371 to 465) that are responsible for NP homo-oligomerization (44–46). The amino acids at positions 286 and 437 are located in these areas (Fig. 6A); therefore, we investigated whether they affect NP-NP homo-oligomerization by using a coimmunoprecipitation assay and Western blotting. To this end, we transfected 293T cells with Myc-tagged NP and Flag-tagged NP. At 36 h posttransfection, cell lysates were immunoprecipitated with an anti-Myc monoclonal antibody (MAb), followed by Western blotting with MAbs against the Myc tag and the Flag tag. As shown in Fig. 6B, the amounts of the four Myc-tagged NPs were comparable, and the amounts of the four coimmunoprecipitated Flag-tagged NPs were also comparable (Fig. 6B). Similar results were obtained in a reverse coimmunoprecipitation experiment that was performed with an anti-Flag MAb (Fig. 6C). These results indicate that the amino acids at positions 286 and 437 do not affect the NP-NP homo-oligomerization.

**FIG 7:**
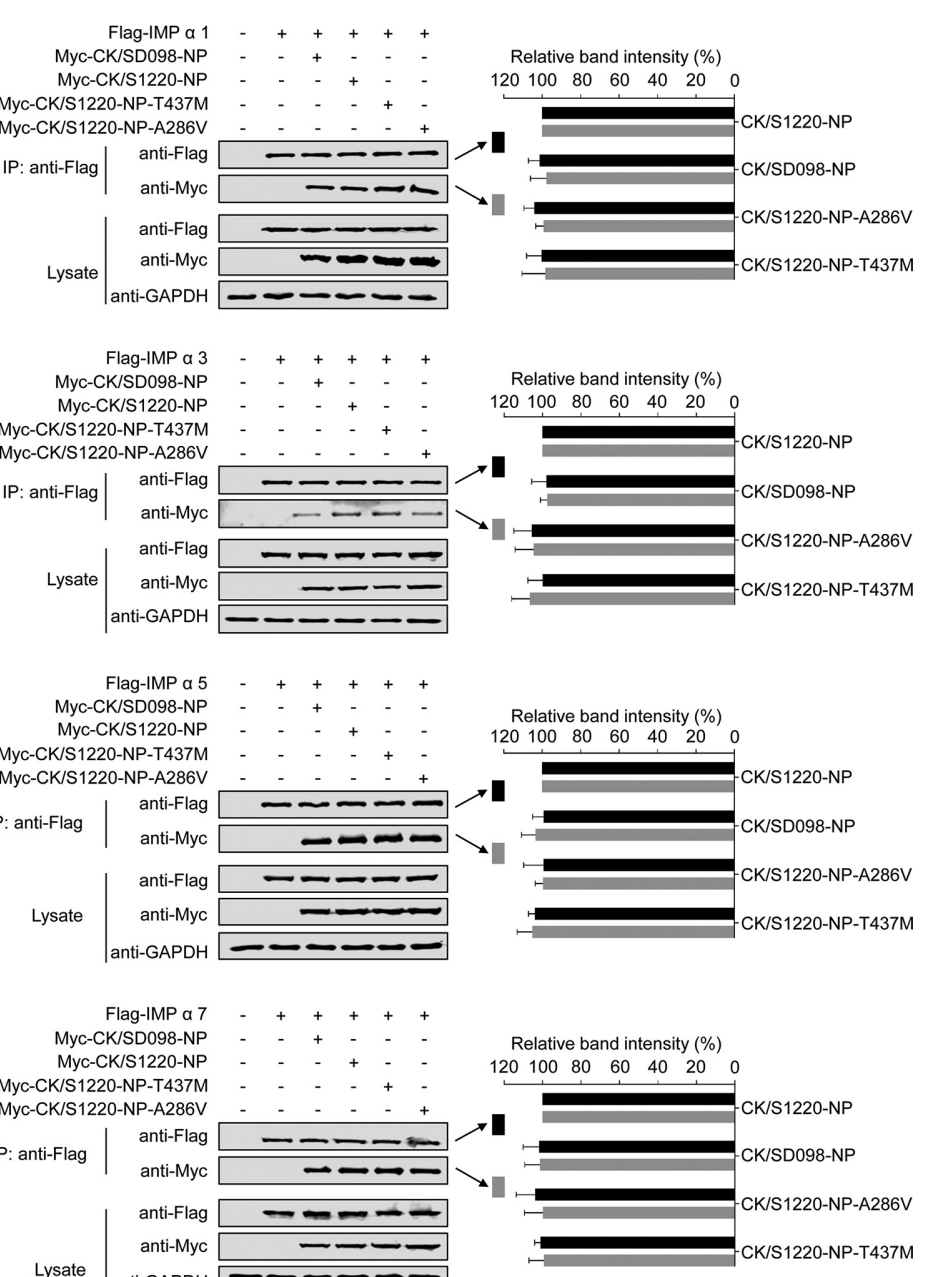
Effects of the amino acid mutations A286V and T437M in NP on the interactions between NP and members of the importin α family. 293T cells were transfected with plasmids expressing Flag-tagged importin α1 (A), importin α3 (B), importin α5 (C), or importin α7 (D) and the indicated Myc-tagged wild-type NP or NP mutants. IP, immunoprecipitation. The band intensities of the Western blots from three assays were quantified by using ImageJ software and compared with the value of the CK/S1220-NP-transfected sample. Statistical analysis was performed by using one-way ANOVA with GraphPad Prism 6 software. When one-way ANOVA was warranted, the *post hoc* analysis was performed using Dunnett’s test for multiple comparisons; no statistically significant differences were detected. Only the band intensity values of the coimmunoprecipitated proteins are shown in the panels.

NP interacts with various isoforms of importin α, including importin α1, α3, α5, and α7, to facilitate vRNP entry into the nucleus during the virus life cycle (47–49). Therefore, we investigated whether the interaction between NP and importin α was affected by the mutations at positions 286 and 437 in NP. To this end, we transfected 293T cells with Flag-tagged importin α and different Myc-tagged NP proteins. At 48 h posttransfection, cell lysates were immunoprecipitated with an anti-Flag MAb and then Western blotted with rabbit MAbs against the Myc tag and the Flag tag to detect importin α and NP, respectively. Flag-tagged importin α1 was coimmunoprecipitated with the various Myc-tagged NP proteins when they were coexpressed in 293T cells (Fig. 7A), indicating that importin α1 interacted with NP proteins. However, the mutations at positions 286 and 437 of NP did not change the interaction of NP with importin α1. Similarly, Flag-tagged importin α3 (Fig. 7B), importin α5 (Fig. 7C), and importin α7 (Fig. 7D) all interacted with Myc-tagged NP proteins, and these interactions were not affected by the mutations at positions 286 and 437 of NP. These results indicate that the mutations at positions 286 and 437 of NP do not affect the interaction of NP with members of the importin α family.

### The A286V and T437M mutations in NP dramatically attenuate the CK/SD008-PB2/627K virus in mice.

We previously reported that the naturally isolated highly pathogenic H7N9 avian influenza virus A/chicken/SD008/2017 (CK/SD008) was not lethal in mice, with an MLD_50_ of >6.5 log_10_ EID_50_, whereas the PB2 627K mutant CK/SD008-PB2/627K was highly lethal in mice, with an MLD_50_ of 1.8 log_10_ EID_50_ (2). To test whether the A286V and T437M mutations in NP could also attenuate the CK/SD008-PB2/627K virus, we rescued CK/SD008-PB2/627K, generated three mutants bearing one or two amino acid changes in the NP protein, and designated them rCK/SD008-PB2/627K, CK/SD008-PB2/627K-NP-A286V, CK/SD008-PB2/627K-NP-T437M, and CK/SD008-PB2/627K-NP-A286V+T437M.

**FIG 8:**
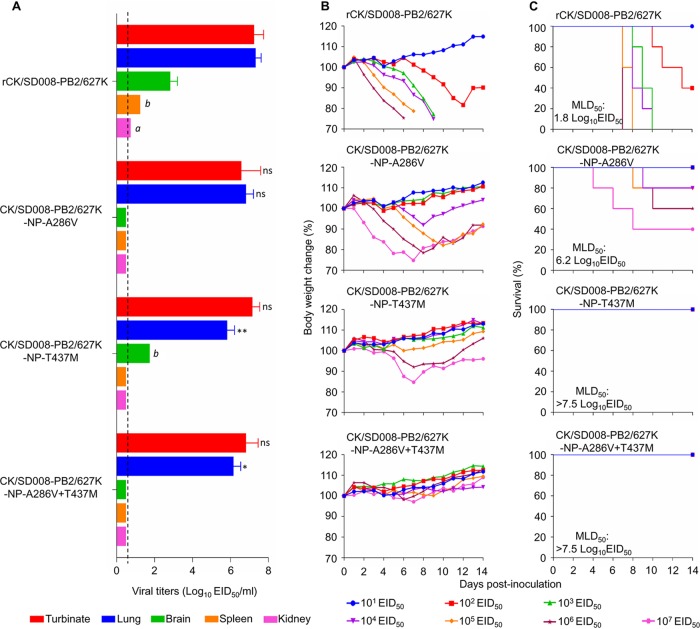
Replication and virulence of H7N9 viruses in BALB/c mice. (A) Virus titers in organs of mice. Data shown are the mean virus titers ± standard deviation (*n* = 3), except for the values labeled *a* and *b*, indicating that virus was detected only in the organs of 1 mouse and 2 mice, respectively. The dashed lines indicate the lower limit of virus detection. The virus titers in other virus-infected mice were compared with those in rCK/SD008-PB2/627K-infected mice, and statistical analysis was performed by using one-way ANOVA with GraphPad Prism 6 software. When one-way ANOVA was warranted, the *post hoc* analysis was performed using Dunnett’s test for multiple comparisons. *, *P* < 0.05; **, *P* < 0.01; ns, not significant. (B) Body weight changes in mice infected with the indicated viruses. (C) MLD_50s_ values of mice infected with the indicated viruses.

The rCK/SD008-PB2/627K virus replicated efficiently in nasal turbinates and lungs, with high titers; low virus titers were detected in the spleens, kidneys, and brains of rCK/SD008-PB2/627K-inoculated mice. The virus was highly lethal in mice, with an MLD_50_ of 1.8 log_10_ EID_50_ (Fig. 8C). The three mutants replicated efficiently in the nasal turbinates and lungs of mice, although the virus titers in the lungs of CK/SD008-PB2/627K-NP-T437M- and CK/SD008-PB2/627K-NP-A286V+T437M-inoculated mice were significantly lower than those in the lungs of rCK/SD008-PB2/627K-inoculated mice (Fig. 8A). The CK/SD008-PB2/627K-NP-T437M virus was also detected in the brains of 2 of the 3 inoculated mice (Fig. 8A). The MLD_50_ of the CK/SD008-PB2/627K-NP-A286V virus was 6.2 log_10_ EID_50_, whereas the MLD_50_ values of the CK/SD008-PB2/627K-NP-T437M and CK/SD008-PB2/627K-NP-A286V+T437M viruses were >7.5 log_10_ EID_50_ (Fig. 8C). These results indicate that the amino acid mutations A286V and T437M in NP also attenuate the virulence of the CK/SD008-PB2/627K virus in mice, and it is reasonable to speculate that these two mutations may attenuate the CK/SD008-PB2/627K virus in a manner similar to that of the CK/S1220 virus, that is, by slowing the nuclear import and export of NP/vRNP and reducing the polymerase activities.

## DISCUSSION

In the present study, we explored the genetic basis for the virulence difference between two H7N9 viruses in mice. The CK/S1220 virus was lethal in mice, with an MLD_50_ of 3.2 log_10_ EID_50_, whereas the CK/SD098 virus was nonlethal in mice, with an MLD_50_ of >7.5 log_10_ EID_50_. Using a loss-of-function strategy, we found that a reassortant bearing the NP of CK/SD098 in the CK/S1220 backbone was attenuated in mice by >10,000-fold, compared with the rCK/S1220 virus. We further identified two amino acid mutations in NP (i.e., A286V and T437M) that synergistically contributed to the attenuation by slowing the nuclear import and export of NP/vRNP in the virus life cycle.

As a major component of the vRNP complex, NP mediates the nuclear import and export of the vRNP complex in the virus life cycle, and the function of NP is affected by various modifications and host factors (36, 47–58). Previous studies identified several amino acids in NP that are involved in the NP posttranslational modifications of ubiquitination, phosphorylation, sumoylation, and acetylation, thereby affecting viral replication or virulence *in vitro* or *in vivo* (51, 52, 54, 55). The two amino acids we identified here are not involved in any of these posttranslational modifications. Several cellular proteins, including members of the importin α family, phospholipid scramblase 1 (PLSCR1), UAP56, and MxA, have been reported to interact with NP (47–49, 53, 56–58). Our coimmunoprecipitation assays indicated that the mutations A286V and T437M in NP did not affect the interaction of NP with members of the importin α family. PLSCR1 interacts with the amino acid 163 to 268 region of NP (56); therefore, the mutations we identified here most likely would not affect the interaction between NP and PLSCR1. It remains to be investigated whether the mutations at positions 286 and 437of NP affect the interactions between NP and other host factors.

The viral proteins encoded by the eight gene segments of influenza A virus play different roles in the viral replication process. Several amino acids in different proteins have been identified as being critical for the function of those proteins. Our previous study indicated that the highly pathogenic H7N9 index strain CK/SD008 was nonlethal in mice (MLD_50_ of >10^6^ EID_50_), even though it bears 286A and 437T in its NP; however, during replication in ferrets, it rapidly acquired the PB2 627K or 701N mutation and then became highly lethal in mice (2). The MLD_50_ values of the CK/S1220-SD098-NP and CK/SD008-PB2/627K-NP-A286V+T437M viruses were >7.5 log_10_ EID_50_, indicating that the mutations A286V and T437M in NP together completely attenuated the naturally isolated virus CK/S1220 and the ferret-adapted virus CK/SD008-PB2/627K in mice, even though the CK/SD008-PB2/627K-NP-A286V+T437M virus bears 627K in its PB2 protein. These data indicate that the newly identified amino acids in NP or the previously identified amino acids in PB2 are required for H7N9 influenza virus lethality in mice but neither of them alone is sufficient to make the H7N9 virus lethal in mice, which confirms that the virulence of influenza virus is a consequence of cooperation among different viral proteins.

Influenza viruses easily mutate during their replication in nature. A series of mammalian-host-adapted mutations in PB2 and HA have been reported to dramatically increase the replication, virulence, or transmissibility of avian influenza viruses in mammals (12–14, 59–66). We compared the NP sequences of 58,747 viruses that are in public databases and we found different amino acids present at positions 286 and 437 of NP, although amino acids 286A and 437T of NP were the predominant residues; 41,193 viruses have 286A and 58,658 viruses have 437T in their NP (see Table S1 in the supplemental material). Among the 58,747 viruses investigated, 41,117 strains have both 286A and 437T in their NP proteins. Viruses bearing 286A and/or 437T in NP were detected from both avian and mammalian hosts, suggesting that 286A and 437T in NP are naturally selected residues rather than mammalian-host-adapted mutations.

In summary, we found that the mutations A286V and T437M collectively attenuated the virulence of H7N9 viruses in mice by slowing the nuclear import and export of NP in the virus life cycle, indicating that the amino acids 286A and 437T in NP are prerequisites for the virulence of H7N9 viruses in mice. Our study further demonstrated that the virulence of influenza virus is a polygenic trait, and these newly identified virulence-related residues in NP may provide new targets for influenza vaccines and antiviral drug development.

## MATERIALS AND METHODS

### Ethics statement and facility.

The present study was carried out in strict accordance with the recommendations in the *Guide for the Care and Use of Laboratory Animals* of the Ministry of Science and Technology of the People’s Republic of China. Studies with highly pathogenic H7N9 avian influenza viruses were conducted in a biosafety level 3+ laboratory approved for such use by the Chinese Ministry of Agriculture. The protocol was approved by the Committee on the Ethics of Animal Experiments of the Harbin Veterinary Research Institute of the Chinese Academy of Agricultural Sciences.

### Cells and viruses.

MDCK cells and HEK 293T cells were grown in Dulbecco’s modified Eagle’s medium supplemented with 6% and 10% fetal bovine serum, respectively, plus antibiotics. A549 cells were grown in nutrient mixture F-12 Ham medium (Kaighn’s modification) with 10% fetal bovine serum. The highly pathogenic H7N9 avian influenza viruses CK/S1220 and CK/SD098 were isolated in 2017 during routine surveillance (30). Virus stocks were propagated in 10-day-old, specific-pathogen-free, embryonated chicken eggs and stored at –70°C.

### Antibodies.

Chicken antisera against A/chicken/Shanghai/S1053/2013(H7N9) and a mouse anti-NP MAb were prepared in our laboratory by using conventional methods. The following primary antibodies were purchased from commercial sources: rabbit anti-glyceraldehyde-3-phosphate dehydrogenase (GAPDH) polyclonal antibody (PAb) (product no. 10494-1-AP from Proteintech, Wuhan, China), mouse anti-Flag MAb (product no. F3165), mouse anti-Myc MAb (product no. M4439), rabbit anti-Flag PAb (product no. F7425), and rabbit anti-Myc PAb (product no. C3965) from Sigma-Aldrich. The secondary antibody used for Western blotting was DyLight 800-conjugated goat anti-rabbit IgG (H+L) (product no. 072-07-15-06), purchased from KPL (Gaithersburg, MD); the secondary antibody used for confocal microscopy was Alexa Fluor 488-conjugated goat anti-mouse IgG (H+L) (product no. A11029), obtained from Thermo Fisher Scientific (Waltham, MA).

### Construction of plasmids.

The eight gene segments of CK/S1220 and CK/SD098 were inserted into the vRNA-mRNA bidirectional transcription vector pBD with a CloneExpress II one-step cloning kit (product no. C112-02; Vazyme). The protein expression plasmids for PB2, PB1, PA, and NP were generated by inserting the gene segments into the pcDNA3.1(+) plasmid (Invitrogen) with the CloneExpress II one-step cloning kit. Mutations were introduced into the NP gene by site-directed mutagenesis (Invitrogen), according to the manufacturer’s protocol. All of the primer sequences used for the construction of plasmids are shown in Table S2 in the supplemental material. All of the constructs were completely sequenced to ensure the absence of unwanted mutations.

### Virus rescue.

Viruses were generated by reverse genetics, as described previously (12). The rescued viruses were confirmed by full-genome sequence analysis, and viruses bearing any unwanted mutations were excluded from further analysis.

### Mouse study.

To evaluate virus replication, groups of 3 mice were lightly anesthetized with CO_2_, inoculated intranasally with 10^6^ EID_50_ of the test virus in a volume of 50 μl, and euthanized on day 3 p.i.; their nasal turbinates, lungs, spleens, kidneys, and brains were collected and titrated for virus infectivity in eggs. To test virulence, groups of 5 mice (Vital River Laboratories, Beijing, China) were lightly anesthetized with CO_2_ and inoculated intranasally with 10-fold serial dilutions containing 10^1^ to 10^6^ EID_50_ or 10^6^ to 10^7^ EID_50_ of H7N9 viruses in a volume of 50 μl. The mice were monitored for 14 days for weight loss and death, and the MLD_50_ values were calculated by using the method described by Reed and Muench (67).

### Luciferase assay of polymerase activity.

293T cells were transfected with pPolI-Luc together with the pRL-TK (Promega) and pcDNA3.1(+) plasmid constructs expressing the polymerase PB2, PB1, PA, and NP genes (100 ng each) plus Lipofectamine 3000 (Invitrogen), as recommended by the manufacturer. pRL-TK is an internal control plasmid, encoding the *Renilla* luciferase protein, to normalize transfection efficiency. Cell extracts were harvested 36 h posttransfection, and luciferase activity was assayed by using the luciferase assay system (Promega). The assay was standardized against the *Renilla* luciferase activity. All experiments were performed in triplicate.

### Confocal microscopy.

A549 cells were grown on glass-bottom dishes and infected with 10^6^ EID_50_ of the indicated viruses. At the indicated times postinfection, the cells were fixed with 4% paraformaldehyde in phosphate-buffered saline (PBS) for 1 h and permeabilized with 0.5% Triton X-100 in PBS for 30 min. After being blocked with 5% bovine serum albumin (BSA) in PBS, the cells were incubated with a mouse anti-NP MAb (1:500) for 2 h. After three washes with PBS, the cells were incubated for 1 h with the secondary antibody [Alexa Fluor 488-conjugated goat anti-mouse IgG (H+L); Thermo Fisher Scientific]. The cells were then washed three times with PBS and incubated with 4ʹ,6-diamidino-2-phenylindole (DAPI) (Thermo Fisher Scientific) for 30 min to stain the nuclei. Cells were observed with a laser scanning confocal microscope (Zeiss). Localization of NP protein was determined by counting the cells (*n = *300) infected with the indicated viruses.

### Viral replication in MDCK cells.

MDCK cells were grown on 12-well plates and infected with the indicated viruses at 10^3^ EID_50_. The inoculum was removed after 1 h of incubation. After three washes with PBS, the cells were incubated with medium containing chicken antiserum against A/chicken/Shanghai/S1053/2013(H7N9) at 37°C for 1 h. The medium was then removed, and the cells were washed three times with PBS. The cells were supplemented with minimal essential medium containing 0.5% BSA and were incubated at 37°C. Virus-containing culture supernatant was collected at 12, 24, and 48 h p.i. and titrated in eggs. The growth kinetics data shown are from three independent experiments.

### Coimmunoprecipitation assay.

293T cells were transfected with the indicated plasmids by using the Lipofectamine 3000 and Plus reagents (Invitrogen, Carlsbad, CA). Cell lysates were prepared at 36 or 48 h posttransfection. Briefly, 293T cells were washed twice with cold PBS, lysed with Pierce immunoprecipitation lysis buffer (Invitrogen) containing complete protease inhibitor cocktail (Roche Diagnostics GmbH, Mannheim, Germany) for 30 min on ice, and then centrifuged at 12,000 rpm at 4°C for 10 min. The supernatants were mixed with the respective primary antibodies, rocked overnight at 4°C, mixed with protein G-agarose beads (Roche), and rocked for 6 to 8 h. The beads were washed four times with PBS containing 1 mM phenylmethylsulfonyl fluoride (PMSF). The bound proteins were then boiled in 2× SDS sample buffer, separated by 10% SDS-PAGE, and detected by Western blotting.

### Western blotting.

Protein samples fractionated by SDS-PAGE were transferred to polyvinylidene fluoride membranes (Merck-Millipore). Membranes blocked with 5% skim milk in PBS were incubated for 1 h at room temperature and then were incubated with diluted primary antibody. After incubation with secondary antibody, the blots were visualized by using an Odyssey CLx infrared imaging system (Li-Cor BioSciences, Lincoln, NE). The band intensities of the Western blots from three assays were quantified by using ImageJ software, and the band intensity values of each gel were compared with the value of the CK/S1220-NP-transfected sample.

### Statistical analysis.

Statistical significance between experimental groups was determined by using the one-tailed unpaired *t* test or one-way analysis of variance (ANOVA) with GraphPad Prism 6 software (GraphPad Software Inc., La Jolla, CA). When one-way ANOVA was warranted, *post hoc* analysis was performed using Dunnett’s test for multiple comparisons.

## Supplementary Material

Supplemental file 1
