# Supplementary material for: Amino Acid Mutations A286V and T437M in the Nucleoprotein Attenuate H7N9 Viruses in Mice
Source: J Virol. 2020 Jan 6;94(2):e01530-19. doi: 10.1128/JVI.01530-19 (PMC6955278; doi:10.1128/JVI.01530-19)
Supplement: Supplemental file 1 [file JVI.01530-19-s0001.pdf]

Table S1. Amino acids at positions 286 and 437 of NP of 58,747 influenza viruses.

Amino acid at position 286 of NP	Strain numbers	Amino acid at position 437 of NP	Strain numbers
A	41193	T	58658
S	17215	I	56
T	200	S	13
V	107	A	9
G	23	N	7
L	4	M	2
P	3	R	1
D	1	V	1
I	1	/	/

TABLE S2. Primers used for pBD cDNA construction and for introducing mutations into the NP gene of the mutant viruses.

Purpose	Primer(s) (5'–3')[a]	
	Forward	Reverse
PB2 amplification	TGCCGGCCAGCAAAAGCAGGTCAAATATATTC	CGGGTTATTAGTAGAAACAAGGTCGTTTTTAAACAATTC
PB1 amplification	TGCCGGCCAGCAAAAGCAGGCAAACCATTTG	CGGGTTATTAGTAGAAACAAGGCATTTTTTCATG
PA amplification	TGCCGGCCAGCGAAAGCAGGTATTGATCC	CGGGTTATTAGTAGAAACAAGGTACTTTTTTGG
HA amplification	TGCCGGCCAGCAAAAGCAGGGGATAC	CGGGTTATTAGTAGAAACAAGGGTG
NP amplification	TGCCGGCCAGCAAAAGCAGGGTAGATAATCACTCA	CGGGTTATTAGTAGAAACAAGGGTATTTTTC
NA amplification	TGCCGGCCAGCAAAAGCAGGGTCGAG	CGGGTTATTAGTAGAAACAAGGGTC
M amplification	TGCCGGCCAGCAAAAGCAGGTAGATGTTTAAAG	CGGGTTATTAGTAGAAACAAGGTAGTTTTTTAC
NS amplification	TGCCGGCCAGCAAAAGCAGGGTGACAAAGAC	CGGGTTATTAGTAGAAACAAGGGTGTTTTTTATC
CK/S1220-NP-A286V	**T**TAGTGGATATGACTTTGAGAGAGAAGGGTACTCC	CAAAGTCATATCCACTA**A**CCACTGCAAGTCCGTAC
CK/S1220-NP-T437M	TAGAA**T**GTCTGACATGAGGACTGAAATCATAAGAA	CTCATGTCAGAC**A**TTCTACCCTCATTATTTCCTGT
CK/SD008-PB2/627K-NP-A286V	**T**TAGTGGATATGACTTTGAGAGAGAAGGGTACTCC	CAAAGTCATATCCACTA**A**CCACTGCAAGCCCGTAC
CK/SD008-PB2/627K-NP-T437M	TAGAA**T**GTCTGACATGAGGACTGAAATCATAAGAA	CTCATGTCAGAC**A**TTCTACCCTCATTATTTCCTGT

[a] The nucleotides that have been changed are underlined and in boldface type.
